# Efficacy of bubaline blood derived fibrin glue in silk ligature-induced acute periodontitis in Wistar rats

**DOI:** 10.14202/vetworld.2021.2602-2612

**Published:** 2021-10-06

**Authors:** Poranee Banyatworakul, Nopadon Pirarat, Sujin Sirisawadi, Thanaphum Osathanon, Chanin Kalpravidh

**Affiliations:** 1 Department of Veterinary Surgery, Faculty of Veterinary Science, Chulalongkorn University, Bangkok, 10330 Thailand; 2 Department of Pathology, Faculty of Veterinary Science, Chulalongkorn University, Bangkok, 10330 Thailand; 3 Biochemistry Unit, Department of Veterinary Physiology, Faculty of Veterinary Science, Chulalongkorn University, Bangkok, 10330 Thailand; 4 Dental Stem Cell Biology Research Unit, Department of Anatomy, Faculty of Dentistry, Chulalongkorn University, Bangkok, 10330 Thailand.

**Keywords:** bubaline, fibrin, inflammation, ligature-induced, periodontitis

## Abstract

**Background and Aim:**

Fibrin forms in the coagulation process, enhancing local hemostatic properties and promoting wound healing. The study aimed to evaluate the efficacy of bubaline-derived fibrin glue in silk ligature-induced periodontitis rats.

**Materials and Methods:**

Bubaline blood–derived fibrin glue was prepared using cryoprecipitation and cryocentrifugation. Periodontitis was induced in rats by placing 5-0 silk ligatures around the mandibular first molars. The animals were divided into two groups: (1) Non-treatment and (2) bubaline fibrin glue–treated groups. Plaque, gingival inflammation, and mobility index were scored on days 1, 7, and 14 after intervention. Histological examinations were performed. The mRNA expression of inflammatory cytokines and growth factors was evaluated using a real-time polymerase chain reaction. Ligature-induced periodontitis was confirmed by the increase in inflammatory cell infiltration as well as histological bone and attachment loss.

**Results:**

Compared to the non-treatment group, bubaline fibrin glue application reduced mononuclear cell infiltration into periodontal tissues corresponding to the reduction of collagen destruction. On days 7 and 14 after intervention, the inflammatory score and histological attachment loss were significantly lower in the bubaline fibrin glue–treated group than in the non-treatment group. A significant reduction in histological bone loss was observed in the treated group on day 7. Bubaline fibrin glue application led to a significant reduction of *Tnfa* and *Il1b* mRNA levels, while an increased expression of *Pdgfa*, *Tgfb1*, and *Il10* was observed compared with the control.

**Conclusion:**

Bubaline fibrin glue could be beneficial in periodontitis treatment aiming to reduce inflammation and delay the progression of periodontal disease.

## Introduction

Periodontitis is well recognized as one of the most common oral diseases in humans and companion animals [1,2]. Eighty percent of dogs show signs of periodontitis [3]. Approximately 20% of humans are affected by periodontal disease [4]. In the USA, 47.2% of adults aged over 30 years suffer from periodontal disease, rising to 68% for people older than 65 years [5-7]. Periodontitis is characterized by periodontal inflammation, clinical attachment loss, and alveolar bone destruction [8]. Periodontitis is a complex infectious disease resulting from interactions between microorganisms and host responses. Environmental factors and genetic susceptibility are modifying factors. Inflammatory processes and immunological responses make a crucial contribution to disease progression [9]. These processes induce the release of cytokines and chemokines, and enhance osteoclast formation and function, ultimately leading to alveolar bone destruction. Hence, the initiation and modulation of these cytokines play a critical role in periodontitis pathogenesis. Conventional scaling and root planning followed by periodontal surgery are utilized to control the progression of the disease. The rate and pattern of the healing process depend on host defense mechanisms and surgical factors [10]. However, the regeneration of functional periodontium remains a challenge. Several adjuvant treatments have been proposed; for example, irrigation with local antiseptic and application of antibodies against the microbe’s virulent factors [11,12]. The use of anti-inflammatory agents has been proposed as another adjuvant approach in periodontitis treatment [13]. For example, the application of orabase cashew gum polysaccharide reduced alveolar bone destruction and inflammatory cytokine expression in a ligature-induced periodontitis model [14], and rice peptide injections inhibited bone loss and reduced osteoclast numbers compared to untreated experimental periodontitis [15]. These could represent therapeutic options to control the progression of periodontal disease and to promote a microenvironment conducive to periodontal regeneration.

Fibrin functions to control hemostasis, create sealing, and facilitate wound healing. Fibrin also contains osteoinductive properties and has been utilized as a component in bone grafts in the oral cavity [16,17]. The utilization of fibrin has been proved to benefit wound healing. It significantly reduces the presence of microorganisms on partial thickness burn wounds *in vivo* [18]. A fibrin adhesive has been implemented for bleeding control and wound closure in the oral cavity as an alternative to conventional suturing [19,20]. Periodontal flap closure using fibrin sealant results in a reduction of inflammatory cell accumulation but an increase in blood vessels in the wound area compared with the use of conventional sutures [21]. The application of fibrin in periodontal surgery reduces interleukin-1β (IL-1β) and IL-8 levels in gingival crevicular fluid [22]. In addition, the bleeding parameter is significantly decreased in those defects treated with fibrin sealant compared with controls [22]. However, the supply of fibrinogen, a substrate for fibrin formation, is limited. Modification of other potential sources of fibrinogen is of interest due to their supply availability and economic benefit. Moreover, in veterinary practice, most cases of periodontitis are in elderly animals with complicated systemic diseases. Anemia is the most common limitation of elderly animals, limiting the preparation of large amounts of autologous fibrin glue. Bubaline blood is proposed as an alternative fibrinogen source. In this respect, bubaline blood exhibits the highest fibrinogen levels from a cryoprecipitate preparation compared with those from bovine and ovine sources [23]. Bubaline-derived fibrin has been reported to promote angiogenesis and reduce skin graft loss in pig surgical wounds [24]. Hence, buffalo blood–derived fibrinogen might be a potential source of fibrin adhesive glue.

The study aimed to investigate the effect of bubaline fibrin glue in rat ligature-induced periodontitis through clinical examination, histopathological evaluation, and inflammatory cytokine expression. The present study seeks to provide an essential *in vivo* understanding of bubaline fibrin glue treatment for periodontal applications.

## Materials and Methods

### Ethical approval

All animal studies were approved by Chulalongkorn University Animal Care and Use Committee (Animal Use Protocol #1531029) and all methods were carried out in accordance with relevant guidelines and regulations.

### Study period and location

The study was conducted from May 2019 to June 2020. The study was conducted at Department of Surgery, Faculty of Veterinary Science, Chulalongkorn University.

### Animals

Thirty-three male Wistar rats (60 days old) were housed in standard cages in an air-conditioned room (23-25°C) at the Laboratory Animal Facility, Faculty of Veterinary Science, Chulalongkorn University, Thailand. Animals were provided with a commercial feed and water *ad libitum*.

### Fibrin preparation

Thai buffalo were used for fibrin preparation. Animals that exhibited zoonotic diseases such as brucellosis, leptospirosis, tuberculosis, bovine viral diarrhea, and trypanosomiasis were excluded from the study. The preparation protocol was adapted according to information from a previous publication, with minor modification [24]. Briefly, fibrin was prepared using blood (450 mL) taken from the jugular vein of buffaloes. The collected blood was preserved in citrate phosphate dextrose (49 mL), and subsequently centrifuged at 1500 rpm at room temperature for 30 min to separate the platelet-rich plasma (PRP) and the packed red cells. The PRP was then divided into two compartments. The first aliquot of PRP (20 mL) was mixed with 2.84 mM citric acid (180 mL) and then cryocentrifuged at 3000 ×*g* at 4°C for 5 min. After discarding the supernatant, the remaining part was mixed with CaCl_2_ (1.14 mL) and NaHCO_3_ (75 mM; 0.7 mL) before clot formation occurred. The thrombin was removed and kept at −80°C. The remaining PRP was mixed with tranexamic acid (3.2 mL) and 99% v/v cold ethanol (12 mL). The mixture was then incubated in an ice water bath at 0°C for 30 min and subsequently cryocentrifuged at 3,000 ×*g* at 0°C for 20 min. The fibrinogen was mixed with 0.9% w/v NaCl (5 mL), then thawed at 37°C, and kept at −80°C. The bubaline fibrin glue was prepared by mixing the fibrinogen and thrombin solutions in a ratio of 5:1 (v/v). The bacterial culture and drug sensitivity of these solutions were assessed for contamination.

### Experimental design and fibrin glue administration

The first animal experiment aimed to confirm the ligature-induced periodontitis model. Rats received ligation of the gingival tissues at the left and right mandibular first molars and were subsequently sacrificed on days 1 and 7 post-ligation (1 DPL and 7 DPL). Animals without ligation were used as the control. Two samples (left and right mandibles) were collected from each rat. Each group consisted of three rats; thus, a total of six mandible samples per group were available for further evaluation. The second experiment sought to evaluate the effect of bubaline blood–derived fibrin glue in ligature-induced periodontitis. Rats received ligation for 7 days. Subsequently, the ligatures were removed, and the intervention was applied. The rats were divided into two groups. In the first group, rats were maintained without any treatment after ligation removal. For the second group, rats received immediate application of the bubaline blood–derived fibrin glue in the periodontal pocket on ligature removal.

### Experimental procedure

Each rat was sedated with intramuscular ketamine (0.08 mL/100 g body weight; Hameln Pharmaceuticals GmbH, Germany) and xylazine HCl (0.04 mL/100 g body weight; Laboratories Calier, S.A, Spain), followed by isoflurane (Baxter^®^, Puerto Rico) mask induction. Rats were oxygenated with 100% oxygen through a gas anesthesia mask and monitored by recording heart rate, respiration rate, and temperature every 5 min. Then, the anesthesia stage was maintained by isoflurane inhalation. A sterile 5-0 black braided silk thread (surgical silk suture; Covidien, Dominican) was ligated subgingivally around the neck of the left and right mandibular first molars [25]. For the bubaline blood–derived fibrin glue–treated group, the glue was inserted into the periodontal pocket using individual syringes of fibrinogen and thrombin at approximately 0.05 mL per syringe. The fibrin glue was allowed to clot completely for 40 s. A dental air-water syringe unit was used to air dry the oral cavity after fibrin glue application.

On 1, 7, and 14 days after intervention, rats were sacrificed and randomly assigned for clinical examination, histological evaluation, and cytokine gene expression analysis (n=3 at each time point). Euthanization was performed with an overdose inhalation of an anesthetic drug (Isoflurane, Baxter®, Puerto Rico). The lower jaws were removed and fixed with 10% neutral buffered formalin for 48 h [26]. Some gingival tissue was cut, washed with sterile RNAse-free water and kept in Trizol reagent (Invitrogen, Carlsbad, CA, USA) at −80°C for further quantitative reverse transcription-polymerase chain reaction (qPCR).

### Clinical examination

Clinical evaluation of the dissected mandible was performed. The plaque index (PI) was recorded on four areas (mesiolingual, distolingual, mesiobuccal, and distobuccal) of each tooth [27]. Scoring criteria were: Score 0: No plaque; score 1: Mild plaque accumulation on the free gingival margin and the surface of the tooth; score 2: Moderate plaque accumulation on the surface of the tooth and deposits in the periodontal pocket; and score 3: Severe plaque accumulation on the gingival margin and abundance in the periodontal pocket.

The intensity of the gingival inflammation was examined on the four areas of the tooth. The color, density, and consistency of the gingiva were evaluated using the gingival index (GI) [27]. Scoring criteria were: Score 0: No inflammation and healthy periodontium; score 1: Mild inflammation with a slight change in the color of the gingiva and no bleeding when probed; score 2: Moderate inflammation with a significant change in the color and consistency of the gingiva, and bleeding when probed; and score 3: Severe inflammation with a significant change in the color, consistency, density of the gingiva, and spontaneous bleeding.

The mobility index (MI) was examined according to the previous publications [28,29]. Scoring criteria were: Score 0: No mobility; score 1: Slightly mobile (buccolingual direction); score 2: Moderate mobility (buccolingual and mesiodistal direction); and score 3: Severe mobility (buccolingual, mesiodistal, and vertical direction).

### Histological analysis

Standard histopathology was performed. After fixation with 10% neutral buffered formalin, the mandibular samples were gently washed and decalcified in 10% nitric acid for 14 days. The samples were further dehydrated in ethanol solution, embedded in paraffin, sectioned at 5 μm in a mesiodistal direction, and stained with hematoxylin and eosin. Sections consisted of the first and second molars, the interproximal alveolar bone crest (BC), and the root pulp chambers. Histological bone loss, histological attachment loss, and inflammation scoring were evaluated. Masson’s trichrome staining was employed to identify collagen fibers.

Histological bone loss was assessed by calculation of the distance (μm) between the cementoenamel junction (CEJ) and alveolar BC. Histological attachment loss was examined by calculating the distance (μm) between the CEJ and periodontal ligament (PL). The interproximal alveolar bone was examined on five slides per sample. The histological assessment was analyzed by two veterinary pathologists in double-blind fashion. These histometric observations were performed with a light microscope (Primo star, Zeiss®, Germany) under 10× magnification. The images were captured (EOS 550D, Canon, Japan) and analyzed (i-Solution™, IMT i-Solution Inc., USA).

Inflammation and fibrosis scoring were evaluated according to previously published criteria [19] under 40× magnification. Score criteria were: Score 0: No inflammatory cells, fibroblasts or fibrocytes; score 1: Inflammatory cells, fibroblasts, and fibrocytes found in 1%–35% of the fields; score 2: Inflammatory cells, fibroblasts, and fibrocytes found in 36%–70% of the fields; and score 3: Inflammatory cells, fibroblasts, and fibrocytes found in over 70% of the fields.

### Cytokine gene expression analysis

The gingival tissues (3 mm×3 mm) were collected from the lingual site of the tooth, using a method described previously [30]. The samples were rinsed with cold sterile phosphate-buffered saline and kept at −80°C. Six samples from each group were collected in each period. RNA isolation with Trizol reagent (Invitrogen, Carlsbad, CA, USA) was done following the manufacturer’s instructions. RNA was purified with RNAse-free DNAse I (Ambion, USA). The purity and the concentration of the RNA samples were measured using a Nanodrop spectrophotometer (Thermo Scientific, USA) after DNAse digestion. Total RNA (1 μg) was employed to effect conversion into complementary DNA using a reverse transcription kit (ImProm-II^™^ Reverse Transcription System, Promega, USA). qPCR was performed in duplicate for each sample using KAPA SYBR^®^ Fast qPCR Kit (Kapa Biosystems, Massachusetts, USA) in a Swift Spectrum™ 48 Real-Time Thermal Cycler (Esco Healthcare, Singapore). The oligonucleotide sequences are shown in Table-1. *Actb* mRNA levels were used as the reference control. The mRNA expression data were calculated using the log_10_ (2^-∆∆Ct^) method.

**Table-1:** The oligonucleotide sequences.

Gene	Forward (5’- 3’)	Reverse (5’- 3’)	Product (bp)	NCBI accession No.
*Il1b*	GACTTCACCATGGAACCCGT	GGAGACTGCCCATTCTCGAC	104	NM_031512
*Tnfa*	CTGTGCCTCAGCCTCTTCTC	ACTGATGAGAGGGAGCCCAT	126	AJ002278
*Il10*	TTGAACCACCCGGCATCTAC	CCAAGGAGTTGCTCCCGTTA	91	NM_012854
*Pdgfa*	GTCAGGGCTAGTGCCCATTT	ACGTCTTGTCTGGGTGATGC	84	NM_022595
*Tgfb1*	CACTCCCGTGGCTTCTAGTG	GGACTGGCGAGCCTTAGTTT	145	NM_021578
*Actb*	TGTTGCCCTAGACTTCGAGCA	GGACCCAGGAAGGAAGGCT	155	NM_031144.3

### Statistical analysis

Data are presented as means±standard error. The unpaired t*-*test was employed for two group comparisons. For three group comparisons, one-way analysis of variance was used, followed by Dunnett’s multiple comparison tests. The statistical analysis was performed using Prism 8 (GraphPad Software, USA). Differences were considered statistically significant when p<0.05.

## Results

### Ligation-induced periodontal tissue inflammation

**Figure-1:**
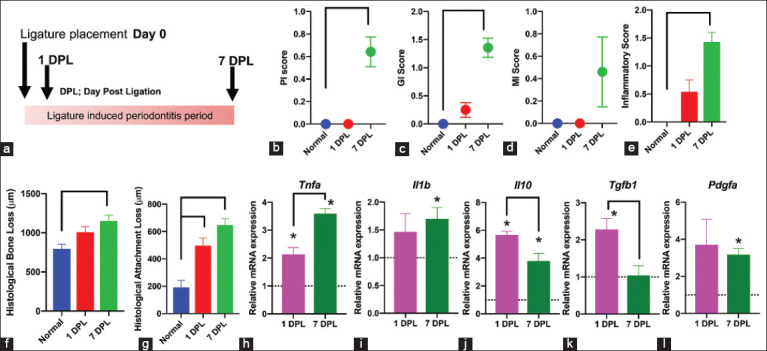
Silk ligation induced periodontitis in rat molars. Animals received silk ligation around mandibular first molars. The diagram of experimental design is demonstrated in (a). On 1 and 7 DPL, plaque index, gingival index, and mobility index were evaluated compared to the controls (b-d). Specimens were collected for histological analysis. Inflammatory score (e), histological bone loss (f), and histological attachment loss (g) were determined. In addition, the mRNA expression of cytokines and growth factors was determined using qPCR (h-l). The dotted line represents the normalized expression levels of the control. Bars indicate a statistically significant difference. Asterisks indicate statistically significant differences compared with the expression levels of the control.

First, the ligature-induced periodontitis model was validated (Figure-1A). On days 1 and 7 after ligation (1 and 7 DPL), clinical parameters, a histometric analysis, and gene expression analyses were performed. Subgingival ligation resulted in a significant increase in PI and GI on 7 DPL compared with the control (p<0.05) (Figures-1B and C). MI was also increased on 7 DPL but there was no statistically significant difference (Figure-1D). In histological analyses, silk ligation markedly induced inflammation, histological bone loss, and histological attachment loss on 7 DPL (p<0.05) (Figures-1E-G). Further, a significant increase in *Tnfa*, *Il1b*, and *Il10* mRNA levels was observed compared with the unligated control (p<0.05) (the control values are presented as dotted lines; Figures-1H-J). *Tnfa* mRNA levels were significantly upregulated on 7 DPL compared to 1 DPL (p<0.05), while the reduction in *Il10* mRNA expression was notable on 7 DPL compared to 1 DPL. *Tgfb1* mRNA levels were significantly higher on 1 DPL compared to the unligated control and marked downregulation was detected on 7 DPL compared to the levels on 1 DPL (p<0.05) (Figure-1K). *Pdgfa* mRNA levels were significantly greater on 7 DPL compared with the unligated control (p<0.05) but there was no significant difference between 7 DPL and 1 PDL (Figure-1L). Taken together, this evidence implies that subgingival silk ligation induces periodontal inflammation and leads to bone and attachment loss in rat molars.

### Effect of bubaline fibrin glue application in ligature-induced periodontitis

**Figure-2:**
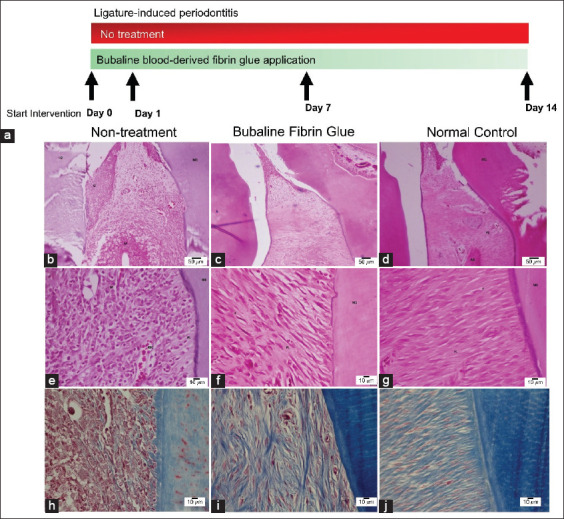
Histological morphology of bubaline fibrin glue treated periodontitis. The diagram of experimental design is shown in (a). Representative images of periodontal tissues of non-treatment (b, e, and h) and bubaline fibrin glue treatment groups (c, f, and i) on 14 days after intervention. The un-ligated samples were used as the normal control (d, g, and j).

Rats received ligation for 7 days. After removal of the ligation, rats were divided into two groups: Non-treatment control and bubaline blood–derived fibrin glue–treated groups. On days 1, 7, and 14 after the intervention, samples were collected for clinical parameters, histometric, and gene expression analysis (Figure-2A).

### Clinical parameter analyses

After the intervention, a decreasing trend in PI score on day 14 after fibrin intervention was observed in both non-treatment and bubaline fibrin glue application groups compared with day 7 (Table-2). However, a significant increase in PI score was noted on day 7 compared with that on day 1 after fibrin intervention in the bubaline fibrin glue–treated groups (p<0.05). There was no significant difference in PI score between the non-treatment control and bubaline fibrin glue–treated groups at all-time points. Similarly, the GI score declined slightly from day 7 to day 14 after fibrin intervention. There was no significant difference in GI score between the non-treatment control and bubaline fibrin glue–treated groups at all-time points. However, the bubaline fibrin glue–treated group exhibited higher GI scores on day 7 compared with day 1 after fibrin intervention (p<0.05). Further, there was no significant difference in the MI between the two groups at any time point.

**Table-2:** Clinical examination parameters.

Group	Time of data collection after intervention		

	1 DPL	7 DPL	14 DPL
Plaque index			
Non-treatment	0.33±0.21	0.17±0.17	0.00±0.00
Bubaline fibrin	0.00±0.00	0.70±0.21*	0.40±0.16
Gingival index			
Non-treatment	0.67±0.21	0.67±0.21	0.50±0.22
Bubaline fibrin	0.38±0.18	1.10±0.18*	0.90±0.10
Mobility index			
Non-treatment	0.00±0.00	0.00±0.00	0.00±0.00
Bubaline fibrin	0.00±0.00	0.10±0.10	0.00±0.00

### Histopathological analyses

**Figure-3:**
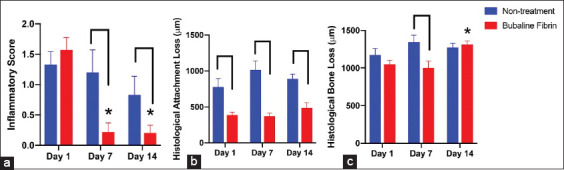
Histological analysis of bubaline fibrin glue treated periodontitis. Inflammatory score (a), histological attachment loss (b), and histological bone loss (c) were examined on 1, 7, and 14 days after intervention. Bars indicate a statistically significant difference. Asterisks indicate the statistically significant differences compared with day 1 after intervention.

Histological observations on day 14 after intervention demonstrated that the non-treatment control group exhibited severe chronic localized granulomatous lesions in the periodontium (Figures-2B, 2E, and 2H). Dramatic mononuclear cell infiltration and PL destruction were observed. In bubaline fibrin glue–treated molars, the periodontium showed mild inflammatory cell infiltration in the gingival epithelium and periodontal tissues (Figures-2C, 2F, and 2I). Mild PL degradation was observed. The PL presented intact collagen bundles. Periodontal tissues in normal condition are illustrated in Figures-2D, 2G, and 2J as a reference for normal tissue morphology. A reduced inflammatory score was demonstrated in a time-dependent manner in both groups (Figure-3A). In bubaline fibrin glue treatment, a significant reduction in the inflammatory score was seen on days 7 and 14 after fibrin intervention compared with the non-treatment control (p<0.05). Moreover, the inflammatory scores of the bubaline fibrin glue–treated group on days 7 and 14 after fibrin intervention were markedly reduced compared to those on day 1 (p<0.05). For histological attachment loss (Figure-3B), the non-treatment group showed a slight increase but there was no statistically significant difference. Histological attachment loss was dramatically reduced in the bubaline fibrin glue–treated group compared with the non-treated control at all-time points (p<0.05). A significant reduction of histological bone loss was noted in the bubaline fibrin–treated group on day 7 after fibrin intervention (p<0.05) (Figure-3C).

### Gene expression analysis

**Figure-4:**
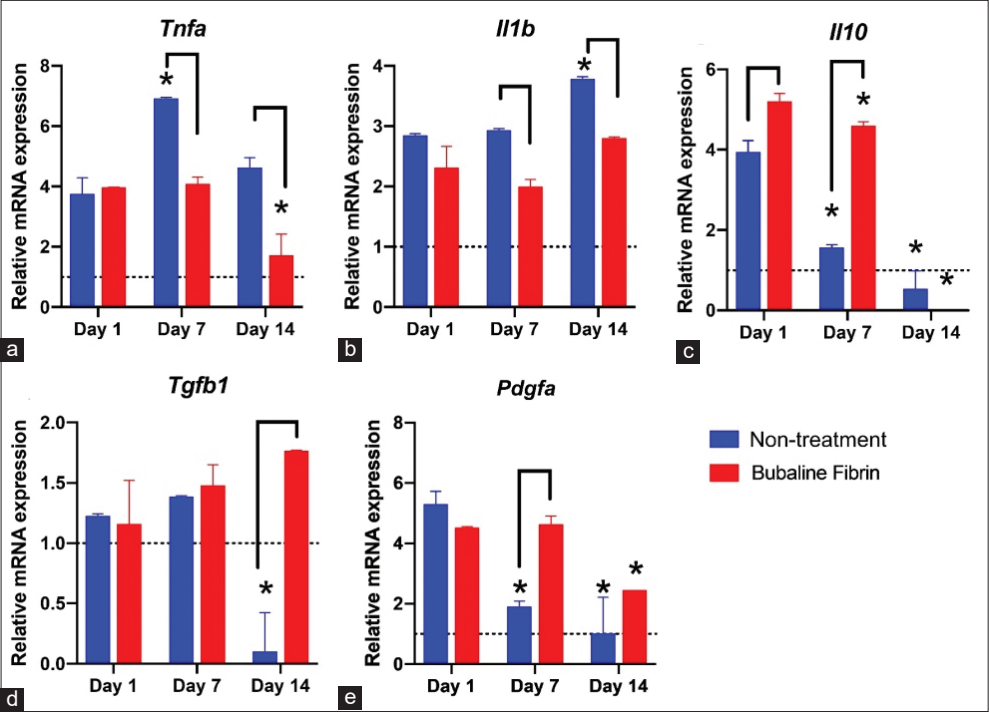
Gene expression analyses of bubaline fibrin glue treated periodontitis. The mRNA expression levels were evaluated using qPCR (a-e). The dot line represented the normalized expression levels of the un-ligated control. Bars indicate a statistically significant difference. Asterisks indicate the statistically significant difference compared with day 1 after intervention.

An upregulation of *Tnfa* and *Il1b* mRNA expression was observed in both non-treated and bubaline fibrin glue–treated groups compared to the unligated control (the unligated control values are presented as dotted lines; Figures-4A and B). However, the mRNA levels of these genes in bubaline fibrin glue–treated conditions were significantly lower than in the non-treatment control on days 7 and 14 after fibrin intervention (p<0.05). In a time-course observation, the mRNA expression of *Tnfa* and *Il1b* was increased in the non-treatment control while the reduction in *Tnfa* and *Il1b* mRNA levels was clearly notable in a time-dependent manner in the bubaline fibrin glue–treated conditions. Expression of *Il10* mRNA was decreased in a time-dependent manner in both non-treated and bubaline fibrin glue–treated groups (Figure-4C). However, the bubaline fibrin glue–treated condition exhibited significantly higher *Il10* mRNA levels compared with the non-treated control (p<0.05). For *Tgfb1* mRNA levels, the non-treatment condition showed decreased expression on day 14 after fibrin intervention compared with the unligated control (dotted line) (Figure-4D). In contrast, bubaline fibrin glue treatment significantly enhanced *Tgfb1* mRNA expression compared with the non-treated group on day 14 after fibrin intervention. Both non-treated and bubaline fibrin glue–treated groups exhibited reduced *Pdgfa* mRNA expression in a time-dependent manner (Figure-4E). However, a dramatic decrease was noted in the non-treatment groups compared with the bubaline fibrin glue–treated condition (p<0.05).

## Discussion

Fibrin is derived from thrombin-cleaved fibrinogen, and subsequently, with the function of factor XIIIa, fibrin crosslinks are formed. Hence, the gel-like substance fibrin is suitable for use and easy to manipulate in a clinical setting. To fabricate fibrinogen hydrogel, protein precipitation is required. Thus, the physical and biological properties of fibrinogen are compromised [31]. Fibrin glue has been employed in various clinical applications, including as a local hemostatic agent, wound sealant, and drug carrier. Fibrin is degraded *in vivo* within 30 days after implantation [32]. Fibrin glue containing thrombin and calcium stimulates the proliferation and collagen synthesis of human dermal fibroblasts [33]. It has also been shown to induce the proliferation and migration of human airway smooth muscle cells and human umbilical cord blood endothelial cells [34,35]. Thrombin preconditioning results in increased extracellular vesicle production from mesenchymal stem cells [34], and these thrombin-induced extracellular vesicles promote full-thickness skin wound healing in rats [34]. Taking all the evidence together, fibrin exhibits superior properties enabling the facilitation of many cellular responses leading to the promotion of wound healing. However, the effect of fibrinogen on periodontal healing requires further investigation.

Various techniques, including inoculation of pathogenic bacteria, dietary intake, and ligation, have been used to induce periodontitis in experimental animals [36-39]. Our study confirmed the efficacy of the silk ligation technique for the induction of periodontitis, as alveolar bone and PL loss were clearly observed in the experimental rats. As in a previous study, the distance between the BC or the alveolar ligament and CEJ was greater in ligated groups than in non-ligated rats [26]. The present study also demonstrated an increase in pro-inflammatory cytokine (*Tnfa*) and a decrease in anti-inflammatory cytokine (*Il10*) mRNA expression on 7 DPL compared to 1 DPL. *Tnfa*, produced by macrophages and monocytes, is an important inflammatory cytokine involved in the pathogenesis of periodontal disease. *Tnfa* induces inflammation through stimulation of other inflammatory mediators. *Tnfa* promotes matrix metalloproteinase production, leading to tissue destruction. Further, *Tnfa* enhances bone resorption by stimulating osteoclast activity and interfering with bone formation [40-42]. *Il10*, an anti-inflammatory cytokine, acts as a protective cytokine in periodontitis. It inhibits the synthesis of pro-inflammatory cytokines, including *Il1*, *Il2*, *Il6*, *Il8*, *Tnfa*, and *Ifng*. Hence, the change in *Tnfa* and *Il10* mRNA expression at later time points after ligation indicates the progression of periodontal tissue inflammation.

The previous studies employing similar fibrin glue preparation protocols have reported that the fibrin was composed of concentrated fibrinogen and growth factors [43]. The enrichment of growth factors, for example, *Pdgf*, *Tgfb*, vascular endothelial growth factor, basic fibroblast growth factor, and epidermal growth factor, not only promotes PDL cell proliferation but also stimulates collagen production [44-46]. Application of a fibrin adhesive in periodontal defects in dogs results in higher new bone formation than that noted in control defects [47]. Taking all the evidence together, fibrin glue could be beneficial in periodontal regeneration. Given the limitation presented by deriving blood samples from aged animal, the use of xenogenic fibrin glue has been proposed. Bubaline fibrin is considered to be bubaline blood as the latter contains a high concentration of fibrinogen. Bubaline-derived fibrin derivatives exhibit excellent biocompatibility to canine PL cells. It promotes cell proliferation and spread as well as enhancing mineralization *in vitro* [48]. The biocompatibility and feasibility of bubaline fibrin glue in pig skin [24], the pig oral cavity, and dog eye (Unpublished data) have been examined.

This study addressed the safety of clinical application of fibrin. Four buffaloes were recruited from a closed environment and were determined to be free from serious zoonotic diseases, such as brucellosis, tuberculosis, rabies, leptospirosis, bovine viral diarrhea, and trypanosomiasis. Routine physical examination and screening of blood samples were performed before experiments.

In the present study, we observed corresponding clinical parameters between PI and GI scores, increases in which were noted in the fibrin–treated group. However, PI and GI scores in the bubaline fibrin–treated group were higher than those in control on 7 and 14 DPL. No significant difference was observed at 14 DPL. We hypothesized that the application of fibrin glue could alter gingival tissue texture leading to an increase in plaque accumulation. Despite the results of the evaluation of PI and GI scores, histological and gene expression analyses demonstrated that the fibrin glue–treated group had less inflammatory cell accumulation and inflammatory cytokine mRNA expression on 14 DPL compared with the control. The role of fibrin glue application in early plaque accumulation and gingival inflammation should be further investigated in detail.

It is noteworthy that the application of bubaline fibrin glue to the periodontal pocket significantly decreased the loss of attachment and inflammatory scores at every time point when compared with the non-treatment group. Fibrin sealant application in periodontal flap closures results in an increase in the number of vessels and in the amount of connective tissue formation [21]. However, the inflammation is decreased compared with the control [21]. Due to the anti-inflammatory properties of Bβ115-42 from fibrin fragments [49], it is hypothesized that fibrin contributes to the attachment of PLs and the promotion of osteogenesis [50]. However, the present study did not observe any marked difference in histological bone loss in the bubaline fibrin glue–treated group compared with the non-treatment control. This observation can be explained by the fact that the alteration of soft tissue is faster than that of hard tissue, which is in line with the normal wound healing of periodontal structures. Hence, longer observation time points should be further investigated for histological bone loss.

However, it should be noted that the present study employed only histometric analysis for bone loss measurement and inflammatory scoring. Some limitations should be noted. First, the angle of tissue sections might have affected the interpretation of the results for both bone loss measurement and inflammatory scoring. Microcomputerized tomographic analysis and assessment of the Defleshed specimens would be required in future studies to confirm the bone loss measurements. Further, immunohistochemistry staining of inflammatory specific markers should be examined to confirm the accumulation of inflammatory related cells in the areas of interest.

The expression of pro-inflammatory cytokines in healthy periodontal tissues has been detected but at lower levels compared to expression in inflamed tissues. This is due to the existence of the stationary state [51]. Pro-inflammatory cytokines, *Tnfa* and *Il1b*, have been shown to initiate bone resorption in periodontal diseases [51,52]. *Tnfa* levels in rats with periodontitis are significantly higher than those of normal rats after 6 weeks of ligature induction [53,54]. The present study revealed that bubaline fibrin glue treatment led to decreased mRNA expression of *Tnfa* and *Il1b* in a time-dependent manner, and a significant difference was noted compared to the non-treatment control. Our results are in accordance with a previous study which demonstrated that the levels of serum *Tnfa* were decreased in tonsillectomy patients treated with commercial fibrin glue [55]. In addition, it has been shown that utilization of fibrin sealant in periodontal flap closure led to decreased *Il1b*protein expression in the treatment sites, corresponding with the lower plaque deposition and gingival bleeding [22].

Although the mechanism of action of bubaline fibrin is unclear, it is postulated that the sealing function and anti-inflammatory effect of the fibrinogen and the thrombin of the bubaline fibrin glue may contribute to this mechanism [49,55]. The components of bubaline fibrin glue are based on a concentrate of bubaline fibrinogen and thrombin. The combination of these two components forms a crosslinked fibrin clot and further affects wound healing. Fibrin-containing thrombin and calcium stimulates fibroblast proliferation and collagen synthesis in the wound healing process in healthy humans [33]. Commercial fibrin glue has been used as an adhesive or a hemostatic agent to promote wound healing after extraction and to control bleeding disorders [19,56].

The present study demonstrated the upregulation of *Pdgfa* and *Tgfb* mRNA levels in the bubaline fibrin glue–treated group. It has been suggested that the wound healing and the anti-inflammatory properties of fibrin accelerate the expression of *Pdgfa* and *Tgfb*. This is related to the collagen synthesis in the remodeling phase of the inflammatory response [57]. *Pdgfa* and *Tgfb* also act as anti-inflammatory cytokines. They stimulate bone formation and inhibit bone resorption [58]. Indeed, the presence of *Tgfb* is associated with the regeneration of periodontal structures. In this regard, *Tgfb* promotes tenocyte differentiation and maturation in human PL stem cells [59]. *Tgfb* and *Pdgfb* promote PL and gingival fibroblast cell proliferation, and contribute to the enhancement of periodontal cell adhesion on the treated root surface [60-62]. *Pdgfa*-gene delivery promotes *c-Myc* and OPN mRNA expression in cementoblasts, implying the effect of *Pdgfa* on cementoblast cell proliferation and extracellular matrix synthesis. The above evidence confirms the positive influence of *Tgfb* and *Pdgfa* in periodontal tissue regeneration. Hence, the upregulation of *Pdgfa* and *Tgfb* mRNA levels in the bubaline fibrin glue–treated groups implies that its application not only controls inflammation but also enhances periodontal regeneration. However, further investigations are required to investigate this assumption.

The relationship of inflammatory cytokines (*Tnfa* and *Il1b*) to TGF-β function has been reported. *Tnfa* and *Il1b* suppress the inductive effect of *Tgfb* on nerve growth factor expression, which contributes to the regeneration of injured neurons [63]. The present study demonstrated the attenuation effect of bubaline fibrin glue on *Tnfa* and *Il1b*, corresponding to the enhancement of *Tgfb* mRNA levels. Hence, the results imply the interaction of pro-inflammatory cytokines and *Tgfb* in bubaline fibrin glue treatment.

One of the limitations in the present study is that only the mRNA expression of inflammatory cytokines and growth factors was examined. The change in mRNA levels may not directly be related to the change in protein levels and functions since post-transcriptional and post-translational processes are involved in the control of protein expression and regulation activity. Hence, the results of this study should be interpreted with caution. Further investigations should be performed into the role of protein expression levels and protein functions in bubaline blood–derived fibrin on periodontal healing.

## Conclusion

The present study demonstrated evidence of decreased periodontal inflammation when applying bubaline fibrin glue in ligature-induced periodontitis. Bubaline fibrin glue induced the mRNA expression of anti-inflammatory cytokines but reduced the mRNA expression of pro-inflammatory cytokines. A reduction of inflammation and a decrease in periodontal destruction in the bubaline fibrin glue–treated group were also noted in the histological analyses. Hence, bubaline fibrin glue could be an alternative material that could be applied to prevent the progress of periodontal diseases. Clinical studies in small animals are needed to further elucidate the mechanisms of periodontal healing by this material.

## Authors’ Contributions

PB: Contributed to data collection, laboratory processing, data interpretation, and manuscript drafting. SS: Extracted bubaline fibrin glue. CK, NP, and TO: Contributed to conceptual design, data analysis/interpretation, and critical manuscript revision. All authors read and approved the final manuscript.
